# Spinal Inhibitory Interneurons: Gatekeepers of Sensorimotor Pathways

**DOI:** 10.3390/ijms22052667

**Published:** 2021-03-06

**Authors:** Nicholas J. Stachowski, Kimberly J. Dougherty

**Affiliations:** Department of Neurobiology and Anatomy, Marion Murray Spinal Cord Research Center, Drexel University College of Medicine, Philadelphia, PA 19129, USA; njs83@drexel.edu

**Keywords:** spinal cord, inhibitory, interneuron, locomotion, sensorimotor integration

## Abstract

The ability to sense and move within an environment are complex functions necessary for the survival of nearly all species. The spinal cord is both the initial entry site for peripheral information and the final output site for motor response, placing spinal circuits as paramount in mediating sensory responses and coordinating movement. This is partly accomplished through the activation of complex spinal microcircuits that gate afferent signals to filter extraneous stimuli from various sensory modalities and determine which signals are transmitted to higher order structures in the CNS and to spinal motor pathways. A mechanistic understanding of how inhibitory interneurons are organized and employed within the spinal cord will provide potential access points for therapeutics targeting inhibitory deficits underlying various pathologies including sensory and movement disorders. Recent studies using transgenic manipulations, neurochemical profiling, and single-cell transcriptomics have identified distinct populations of inhibitory interneurons which express an array of genetic and/or neurochemical markers that constitute functional microcircuits. In this review, we provide an overview of identified neural components that make up inhibitory microcircuits within the dorsal and ventral spinal cord and highlight the importance of inhibitory control of sensorimotor pathways at the spinal level.

## 1. Introduction

The spinal cord is capable of generating a range of motor behaviors in response to various interoceptive and environmental cues. Somatosensory pathways span the entirety of the neuraxis and recruit a unique combination of neuronal networks to elicit appropriate behavioral responses [1,2,3]. Despite this complexity, central terminals of primary sensory neurons recruited by peripheral stimulation and motor neurons activating the behavioral response co-exist within the spinal cord. Although monosynaptic connections exist between afferents and both motor neurons and projection neurons with supraspinal targets, most responses to sensory stimuli result from integration of multiple modalities of information by networks of neurons throughout the spinal cord before reaching motor neurons or higher order centers [4,5,6,7]. These networks consist of excitatory and inhibitory interneurons (INs) that serve to select for relevant afferent input and prevent aberrant sensory signaling throughout somatosensory pathways [8,9,10]. In order to filter the ongoing, innocuous stimuli from salient cues and select an appropriate behavioral response, sensorimotor pathways are subject to both tonic and discrete inhibitory gating mechanisms. Therefore, the spinal circuits involved in gating nociceptive, proprioceptive, and mechanical signals are optimally located to modulate the activity at both the input and output stages. The complex role of inhibitory INs in afferent gating was established through the seminal work of Melzack and Wall in 1965 and the identification of populations involved has warranted continuous investigation to date [11]. All of the inhibitory circuits discussed herein receive excitatory drive either directly or indirectly from the central terminals of primary afferent fibers. These circuits act to regulate the level of activity within last order output neurons through both direct and indirect mechanisms [12,13,14].

A variety of experimental approaches, including classic electrophysiology and recent complex genetic and transcriptomic techniques, have been used to dissect inhibitory microcircuits [15,16,17,18,19,20]. These studies have determined the anatomical location and neurochemical and/or molecular profile of neural components comprising various functional spinal circuits. This review will examine the most recent characterizations of the inhibitory neural elements within the dorsal and ventral horns and provide examples of the behaviors which they have been shown to mediate.

## 2. Excitatory Input to Sensorimotor Pathways

**Figure 1:**
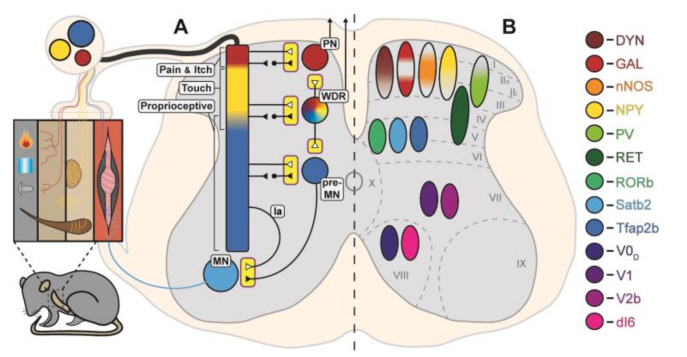
Organization and points of control within sensorimotor pathways. (**A**) Cutaneous and proprioceptive receptors responding to a diverse array of sensory signals terminate in various, and often overlapping, laminae of the spinal cord. Projection neurons (PN) and motor neurons (MN) are the major output neurons of the spinal cord and wide dynamic range (WDR) and pre-motor neurons (pre-MN) neurons play an integrative role in sensorimotor pathways. All of these neurons receive direct (white triangles) and indirect (black triangles) afferent input and are subject to pre- and postsynaptic inhibition (yellow boxes). (**B**) Schematic representation of inhibitory populations involved in afferent gating. Inhibitory interneurons in the superficial dorsal horn are neurochemically subdivided into largely non-overlapping subpopulations, with the exception of dynorphin (DYN) and galanin (GAL) which are highly co-expressed. Molecular markers subdivide inhibitory populations in the deep dorsal and ventral horns. Ovals denote predominant laminar distribution but do not necessarily reflect medial-lateral positioning. Shading within ovals represents the regions of highest density for individual populations.

Primary sensory neurons are excitatory, pseudo-unipolar neurons with cell bodies located in the dorsal root ganglion (DRG), peripheral receptors in the skin and muscles, and central terminals that are concentrated in various layers of the dorsal horn and ventral horn [3,21,22]. Fiber types are broadly divided into major classes based on diameter, threshold for activation, and conduction velocity [23,24,25]. Myelinated, type I, type II, and Aβ afferents are large diameter fibers which rapidly conduct proprioceptive and innocuous mechanosensory signals [26,27,28,29]. Small-diameter, unmyelinated C fibers and medium-diameter, myelinated Aδ fibers are recruited by mechanical, thermal, and/or chemical nociceptive stimuli at either high- or low-threshold intensities, respectively [30,31]. The central terminals from each fiber type are concentrated in distinct laminae of the superficial, deep dorsal, and/or ventral horn (Figure 1A). Intrinsic and anatomical features help to separate fiber types; however, these markers are broad classifications that often label multiple fiber types and do not sufficiently account for the diversity of stimuli.

More recently, afferent fiber types that convey unique sensory input towards spinal effector neurons and higher order centers have been separated using various cell-type specific markers [32]. Unmyelinated C fibers can be divided into peptide expressing and non-peptidergic populations, which transmit mechanical and thermal signals to superficial dorsal horn [33,34,35,36]. The expression of vesicular glutamate transporter 3 (vGluT3) describes an additional subtype of unmyelinated C fibers that are activated by low-threshold mechanical stimuli which share the same laminar target as the previous fiber types (I/II_i_) but none of the neurochemical markers [37,38]. Low-threshold, cutaneous mechanoreceptive (LTMR) afferent fibers terminating throughout laminae II_i_-IV have been identified and described using a combination of molecular profiling [21,39,40], intersectional genetic techniques [8], and selective physiological manipulations [41,42] in mice. Myelinated afferents expressing parvalbumin and vGluT1 target the deep dorsal and ventral horns and are primarily involved in proprioceptive pathways [43,44,45]. These include group Ia and II muscle spindle afferents and group Ib Golgi tendon organs. Identities of molecular markers to separate these muscle afferents have just recently been determined [46,47].

Spinal INs are primary targets for many types of afferent fibers [8,9,48,49]. Much attention has been paid to molecularly defined excitatory IN populations in the dorsal horn and their roles in conveying and amplifying sensations of touch, itch, pain, and position [9,49,50,51,52,53,54]. The majority of these neurons are interposed in pathways between distinct afferent fiber types and ascending tract neurons [13,55,56]. Although often studied in context of sensation, all of these sensations are identified by a motor response, suggesting dynamic interactions across both dorsal and ventral interneuronal circuits within sensorimotor pathways [57,58,59].

In addition to INs which may be more specialized in function, there are several populations of spinal neurons serving a more integrative function. These tend to be located within the deep dorsal horn (III-VI) and include neurons that receive polymodal input from various sensory pathways [60,61,62]. These populations include both ascending tract neurons and INs which are likely to be involved in activating or modulating motor responses [5,44,63]. For example, antenna neurons in laminae III-IV are wide dynamic range neurons receiving monosynaptic input from both Aβ and Aδ afferent fibers and integrate low threshold mechanical signals across sensory pathways [61]. Motor synergy encoder neurons, located in the medial deep dorsal horn, play a similar role within sensorimotor pathways [44]. They receive both descending cortical and proprioceptive afferent input and form monosynaptic contacts onto premotor neurons and motor neurons [44]. The diversity of convergent excitatory drive onto these integrative populations highlights the need for inhibitory gating mechanisms to select context-relevant stimuli in order to elicit the appropriate behavioral response [13,59,64].

Ventral horn INs have been largely studied in the context of locomotion, with distinct populations contributing to rhythm and pattern [65,66,67,68,69]. Locomotor network activity can be initiated and modulated by sensory feedback [70,71,72]. For example, stretch of hip extensor muscles during late stance facilitates the transition to the swing phase [73]. Additionally, when the dorsal part of the paw encounters an obstacle, the limb is lifted higher in order to clear it [74,75]. Thus, both proprioceptors and cutaneous mechanoreceptors influence the activity of locomotor circuits.

There are multiple potential points of control including the afferent and excitatory INs involved in specific pathways, excitatory integrator neurons, locomotor circuit neurons, ascending tract neurons, and motor neurons. Inhibitory neurons reduce the activity in these pathways by postsynaptic and presynaptic inhibitory mechanisms.

## 3. Spinal Modes of Inhibition

Inhibition at the level of the spinal cord maintains appropriate levels of activity within neuronal circuits by regulating sensory information en route to ascending and motor output pathways. Inhibitory INs regulate activity of both excitatory and inhibitory neurons postsynaptically through axo-somatic and/or axo-dendritic synapses [12,13,76]. Evidence for feed-forward inhibitory circuits within sensorimotor pathways is ubiquitous and likely involves afferent input from all fiber types [32,76,77]. Inhibitory neurons in the spinal cord may be glycinergic and/or GABAergic [76]. Dorsal inhibitory neurons largely co-express markers for GABAergic and glycinergic transmission [78,79], whereas ventral inhibitory neurons are mainly glycinergic beyond early postnatal development [76,80,81]. There are many exceptions to this and, in some cases, the expression of GABAergic and glycinergic markers, alone or in combination, can be used to differentiate discrete populations [82,83,84].

Inhibitory synapses onto afferent terminals provide an additional mode of inhibitory gating of sensory information which decreases the excitatory drive to postsynaptic neurons rather than directly depressing the level of activity at the postsynaptic neuron [12,76]. Inhibitory INs mediating presynaptic inhibition are GABAergic [85,86] and are further defined by the expression of GAD65 [43,87,88]. Afferent fibers activate spinal excitatory INs that target GABAergic neurons. These GABAergic neurons form axo-axonic synapses onto afferent terminals. Since intracellular chloride concentrations of afferent terminals are high, GABA binding to cognate GABA_A_ receptors on the terminals results in chloride efflux, producing depolarization. This primary afferent depolarization (PAD) retrogradely invades the afferent fibers, inactivating voltage-gated sodium and/or calcium channels, shunting subsequent afferent signals [76,86,89]. Detailed explanations of various other mechanisms and modulators involved in PAD have been reviewed elsewhere [12,14,86,89].

Developmental genetic studies have determined the transcriptional regulatory framework that underlies inhibitory cell-type diversification throughout the spinal cord [90,91]. All spinal inhibitory neurons express Pax2 throughout early developmental stages and into adulthood [92,93,94,95] but separate transcriptional regulators underlie cell-type specification [17]. In order to narrow down large inhibitory populations to smaller subsets to study specific function, markers that are largely non-overlapping are used and the type of marker, i.e., transcription factor, neuropeptide, calcium binding protein, depends on the region. Populations of inhibitory neurons in the dorsal horn are largely defined by neurochemical markers [18,96] and those in the ventral horn are typically defined by combinations of developmental transcription factors and calcium binding protein expression [43,97]. More recent selective manipulations of these populations provide functional correlates which can be further integrated with anatomical and physiological attributes defined with classic and more recent techniques to establish circuit connectivity. The sections that follow will highlight populations of inhibitory INs that have been shown to regulate sensory-evoked behavioral responses, such as nocifensive reflexes and locomotion, and summarize their defining features, physiology and circuitry (Figure 1B).

## 4. Inhibitory Interneurons in Superficial Dorsal Horn

Inhibitory neuronal populations in laminae I–III of the dorsal horn have the potential to ameliorate nociceptive signaling at the spinal level prior to ascending to result in pain perception [13,98]. Therefore, these neurons have received significant attention and have been well characterized. Four neurochemically defined inhibitory populations, parvalbumin, galanin, nitric oxide synthase, and neuropeptide Y, constitute inhibitory neurons in laminae I-III [18,96]. These four populations make up ~75% of inhibitory neurons in the superficial dorsal horn of mouse, with the remaining inhibitory neurons expressing calretinin [18]. Importantly, most of the markers are not exclusive to inhibitory neurons but are also expressed in excitatory INs to various degrees.

### 4.1. Parvalbumin INs

Parvalbumin (PV) INs in the superficial dorsal horn are primarily located in laminae IIi-III [99,100,101,102], where the majority (>70%) of them are inhibitory [8,18], co-expressing GABA and glycine [103,104]. PV INs have dendrites that extend in the rostral-caudal plane [99] and receive input from myelinated LTMR afferent fibers [101,105]. Their axons are largely restricted to laminae II-III where they target INs in lamina II, including other PV INs and excitatory vertical cells and PKCγ INs [102,105]. In addition to their role in postsynaptic inhibition, PV INs synapse on central terminals of myelinated Aβ and Aδ afferent fibers in laminae II_i_/III [8,99,101,104,105,106], making up a significant portion of axo-axonic synapses mediating presynaptic inhibition of cutaneous mechanical sensory input in this region. Inhibitory PV INs are found in inhibitory synaptic triads, with the same PV IN terminal contacting both central terminals and postsynaptic dendrites [87,101,105,107].

Genetic removal or synaptic silencing of the entire PV IN population leads to allodynia of previously innocuous stimuli [102]. At least part of this effect is thought to result from the loss of inhibition of lamina II excitatory INs, allowing for innocuous signal propagation from low threshold afferents to lamina I nociceptive projection neurons via lamina II excitatory INs, leading to mechanical hypersensitivity [102,105]. PV neurons are also under glycinergic inhibitory control [108] which may provide an intrinsic circuit mechanism for similar disinhibition. Conversely to the effects of genetic removal, activating PV INs ameliorates mechanical hypersensitivity in a mouse nerve injury model [102]. Taken together, these findings indicate that PV INs gate mechanical pain. However, it remains to be determined whether these effects are due to pre- and/or post-synaptic mechanisms.

### 4.2. Galanin and Dynorphin INs

There is extensive co-expression of galanin and dynorphin in the superficial dorsal horn and these neurons constitute a subset of inhibitory INs distinct from PV [18,96,100,109,110]. Galanin and dynorphin INs are primarily located in laminae I–II_o_ with fewer cells in laminae II_i_–III [100,109]. Galanin INs are entirely GABAergic and do not express glycinergic markers [111]; however, dynorphin INs also include excitatory neurons, particularly in the medial aspect of laminae I and II_i_ [109,112]. Immature dynorphin INs receive predominantly C fiber input but by adulthood, they receive multimodal afferent input [113,114]. Axonal arborizations of dynorphin neurons are small and remain within the superficial dorsal horn [100,109], targeting excitatory neurons which are critical components of classical pain pathways, including somatostatin-expressing neurons [113] and gastrin-releasing peptide receptor (GRPR)-expressing INs [110,112,115], as well as lamina I spinoparabrachial neurons [56,114].

Several additional lines of evidence indicate a role for galanin and inhibitory dynorphin INs in gating LTMR afferent input by suppressing the activity of excitatory output neurons in pathways, signaling mechanical pain and itch. Chemogenetic activation of dynorphin INs attenuates responses to pruritogens [112]. Further, somatostatin inhibits dynorphin INs via actions at sst2A receptors, disinhibiting the pathway and potentiating itch [112]. Similarly, activation of galanin INs reduces itch in response to pruritogens and, conversely, genetic ablation of galanin INs leads to an enhancement in itch responses, without affecting nociceptive responses [115]. At the circuit level, the dynorphin and galanin IN activation effects are due to an inhibition of GRPR-expressing excitatory INs which are involved in itch pathways [112,115,116].

Interestingly, genetic ablation of dynorphin INs does not affect itch but instead facilitates Aβ-evoked input to superficial pain pathways and leads to the development of mechanical allodynia [113]. The discrepancies between the activation and dynorphin IN ablation studies may be due to differences in the exact neuronal population targeted. Excitatory INs in deeper laminae (III-IV) express dynorphin and these neurons may be differentially affected [18,109]. Additionally, genetic ablation of dynorphin INs occurred early in development compared to injection of conditional DREADD for activation of adult neurons [112]. This suggests that there may be distinct subpopulations of inhibitory dynorphin/galanin INs providing inhibition of itch and inhibition of innocuous mechanical input that can activate pain circuits.

### 4.3. nNOS INs

Another grouping of inhibitory INs in the superficial dorsal horn primarily expresses nitric oxide synthase (nNOS) [10,18]. There is a limited degree of overlap between the nNOS and dynorphin/galanin populations (~20%) [110,112] and no overlap with the PV population [18]. All nNOS INs in lamina I are GABAergic, however, the majority (~66%) of nNOS INs in lamina II and roughly half of those in lamina III are excitatory INs [96,109]. GABAergic nNOS INs are innervated by unmyelinated or thinly myelinated afferent fibers [117], including nociceptors lacking the capsaicin receptor TRVP1 [118]. Inhibitory axonal terminals from nNOS INs are concentrated in laminae II-III, forming axo-somatic or axo-dendritic synapses; thereby mediating postsynaptic inhibition [117,118]. nNOS terminals make contacts with lamina I giant cells and excitatory PKCy INs in laminae II and III, although to a very limited extent [119,120]. Activation of nNOS INs suppresses responses to mechanical and thermal stimuli [112] and has also been shown to induce spontaneous scratching in the absence of pruritogens [115]. The discrepancies in these studies may be due to concomitant activation of excitatory nNOS neurons which activate GRPR-expressing neurons and lead to itch [115].

### 4.4. Neuropeptide Y (NPY) INs

Expression of NPY distinguishes an additional population of dorsal inhibitory INs in both rat and mouse [18,96,109,121] and has been implicated in pain and itch pathways. NPY INs rarely co-express PV, nNOS, dynorphin and galanin [18,110,121]. All NPY INs in laminae I-III are GABAergic but excitatory NPY INs are found in deeper laminae (IV-VI) [18,121]. The majority of NPY INs receive multimodal input from LTMRs [122,123] and C-fibers that lack TRPV1, conveying noxious mechanical stimuli [118,121]. Inhibitory NPY INs are concentrated in laminae II-III with variable morphology and dense axonal bundles associated with projection neurons in both superficial (I-II) and deep (III-IV) dorsal horn [18,55,121]. Postsynaptic targets include NPY1R-expressing INs [124], lamina II excitatory INs expressing Urocortin (Ucn3) [52,124], and NK1R-expressing projection neurons in laminae I and III [55,121,123,125]. Genetic ablation or chemogenetic silencing of NPY INs leads to excessive responses to mechanical itch but not chemical itch or pain [52,122].

### 4.5. Calretinin INs

Calretinin INs represent a large portion of inhibitory INs in I-II that were not labeled by any of the previously labeled neurochemical markers [18,126]. They are found throughout all laminae, except lamina IX, of the spinal cord but are most densely found in lamina II [126,127]. Only ~15% of calretinin neurons in the superficial laminae are inhibitory and can be distinguished by Pax2 expression, islet cell morphology, and tonic or initial burst firing [126]. They also express substance P [128]. The inhibitory calretinin neurons can be divided into two groups based on sst2A expression [126]. All calretinin neurons respond to noxious stimulation [126]. Inhibitory inputs to inhibitory calretinin INs are predominantly glycinergic and these neurons are inhibited by enkephalin [129]. Since the inhibitory and excitatory calretinin neurons are intermingled, intersectional strategies will be necessary to determine their function.

## 5. Inhibitory Interneurons in Deep Dorsal Horn

The inhibitory populations in the deep dorsal horn receive multimodal afferent input from a wide array of LTMR afferents [8,48]. Importantly, deep dorsal circuits integrating cutaneous afferent transmission not only enable high fidelity sensory discrimination but have also been shown to regulate motor activity [7,50,130,131]. Tactile input will stimulate peripheral sensory receptors and suppress cutaneous afferents through classical gate control, allowing for heterotypic modulation of sensorimotor pathways [11,59,130,131]. The inhibitory INs in this region are well positioned to mediate sensorimotor integration through contacts onto LTMR pathway components as well as ventrally directed proprioceptive afferents, excitatory premotor INs, and ascending pathways [7,8,43,60,125]. Inhibitory neurons in the deep dorsal horn have been identified using molecular screens and have been shown to be involved in presynaptic inhibition and are interposed between afferent fibers and ascending pathways and/or motor neurons, which will be summarized in the following sections.

### 5.1. Early RET + INs

A population of neurons in the laminae III-V expresses receptor tyrosine kinase Ret (RET) during neonatal development and defines a predominantly inhibitory subpopulation that has interesting circuit properties [132]. Early RET INs do not include more superficial neurons which express RET due to later Ret expression [132]. Deep dorsal RET (RET) INs co-express markers for GABAergic and glycinergic neurotransmission and minimally overlap with the PV INs but none of the other neurochemical subpopulations [18,132]. RET INs receive both Aβ, Aδ, and both peptidergic and non-peptidergic C fiber input and polysynaptic inhibitory input from Aβ and C fiber afferents [132]. Downstream targets of RET INs include excitatory PKCγ INs which forward low threshold touch information to projection pathways and somatostatin INs involved in mechanical pain, as well as other inhibitory RET INs [132]. Although postsynaptic responses to RET IN activation are glycinergic, RET boutons are also adjacent to myelinated afferent terminals and those RET terminals contain GAD markers [132], as seen with INs mediating presynaptic inhibition [43,86]. Taken together, these findings indicate RET INs play a role in pre- and postsynaptic inhibition of individual neurons. Further, targeted ablation of the RET IN population induces mechanical and thermal hyperalgesia and exacerbates sensory deficits in models of inflammatory and neuropathic pain [132]. Despite minimal overlap, RET INs share several features with the PV INs described in the previous section and play a critical role in mediating crosstalk between pain and touch pathways.

### 5.2. Rorβ INs

Gene expression profiling and single-cell RNA sequencing have converged on cell-specific markers that define a subset of neurons in the dorsal horn expressing the retinoid-related orphan receptor-β (Rorβ) [8,19]. Rorβ INs are a heterogeneous population in terms of transmitter phenotype, with ~60% being GABAergic, and topographical location divides them into two distinct populations, in lamina III and medially in laminae IV/V [8,133]. The Rorβ INs show minimal overlap with other neurochemically defined inhibitory populations in either region [8,18]. The more superficial population can be divided into neurons that express either glutamatergic markers or both GAD isoforms, and their functional role is not clear [8,133]. However, the deeper population (IV-V) expresses GAD65 which defines GABAergic neurons involved in presynaptic inhibition [43,133]. Genetic knockout of Rorβ results in mice with what was described as a ‘duck-like gait’, in addition to other abnormalities [133,134]. Intersectional genetic studies targeting removal of Rorβ from spinal inhibitory INs or ablation of spinal Rorβ INs elicits similar motor deficits, characterized by pronounced hyperflexion during the swing phase of locomotion [133]. In vitro electrophysiology experiments demonstrate that these mutants have a decreased afferent-evoked PAD amplitude and decreased threshold for sensory-evoked reflexes, indicating a loss of presynaptic inhibitory tone [133]. Genetic manipulations do not affect withdrawal responses to tactile stimuli; however, blocking afferent transmission in the peripheral muscle nerve reduces the hyperflexion gait seen in the mutants [133], suggesting that Rorβ INs gate proprioceptive sensory input to motor pathways.

### 5.3. Satb2 INs

Expression of the nuclear organization factor Satb2 regulates sensorimotor circuit organization in the spinal cord and labels a unique population of neurons that are concentrated in laminae V-VI [44,48,135]. Expression of Satb2 is found in both excitatory and inhibitory INs that are largely topographically divided in the dorsal and ventral aspects of the region, respectively, and depending on genetic strategy, a predominant inhibitory Satb2 IN population can be preferentially identified [44,135]. The Satb2 IN population has axonal terminals extending in a diagonal band across laminae V-VIII and IX, directly contacting individual motor neurons and premotor INs [135]. They constitute a significant portion (~20%) of the deep dorsal premotor neurons, collectively shown to be a population of neurons involved in coordinating motor neuron recruitment across motor pools and, therefore, referred to as motor synergy encoder neurons [44]. Inhibitory Satb2 INs are further anatomically and molecularly divided along the mediolateral axis of lamina V, wherein medial Satb2 INs co-express Ctip2. The Satb2 INs receive direct proprioceptive afferent input, and this is to a greater degree in the medial Ctip+ INs, compared to the lateral Ctip2- INs [135]. Additionally, premotor Satb2 INs are activated by noxious stimulation of the hind paw, indicating that in addition to proprioceptive input, they are polysynaptic targets of nociceptors and a likely site of multimodal convergence in sensorimotor pathways [135]. Satb2 mutants respond to noxious thermal and mechanical stimulation with an exaggerated hyperflexion and aberrant hyperflexion of the ankle was visible during the swing phase of locomotion [135], suggesting that these neurons gate flexion withdrawal reflexes. It is likely that Satb2 INs serve an integrative function and are interposed in polysynaptic, sensorimotor pathways considering their multimodal afferent input and expansive intersegmental innervation of premotor neurons [7,44,135].

### 5.4. Tfap2b INs

Tfap2b INs are largely GABAergic neurons found medially in laminae IV, V, and VI [44]. These neurons receive input from proprioceptive afferents [44]. Where a portion of them likely mediate presynaptic inhibition, they are also premotor INs and are thought to be involved in encoding of motor synergies [44] but have not yet been targeted for genetic manipulation.

## 6. Inhibitory Interneurons in Ventral Horn

Motor circuitry, and minimally motor neurons, must be recruited for sensory-evoked action. Motor neurons are the final output of the nervous system [136] and receive both direct proprioceptive (i.e., Ia) and indirect proprioceptive and cutaneous input from upstream integrative networks [7,15,43]. Proprioceptive afferent input is essential for coordinated movements [137] and produces phase dependent modulation of motor activity, such as during locomotion [71,131]. Cutaneous input may not be essential for overground locomotion, but the disruption of cutaneous sensory pathways leads to deficits when walking over uneven terrain and recovery of locomotor function after spinal cord injury [50,59,138,139]. Where some reflex pathways are depressed during locomotion [140,141], others elicit different motor output based on the position of the affected limb [71,75,142]. Task-dependent gating of reflexes and inhibition of antagonist muscle groups ensures proper motor control and may occur due to pre- or postsynaptic inhibition [86,141]. Phase- and task-dependent modulation of motor output are mediated by ventral inhibitory INs which are essential for coordinated locomotion and are accessed by proprioceptive afferents to refine and adapt motor pattern [17,69,72].

Inhibitory INs in the ventral horn are often divided into four cardinal classes (V0–V3) based on the developmental expression of transcription factors unique to INs derived from the same progenitor domain [69,143]. Less is known regarding the sensory innervation of many of the inhibitory populations identified in this way; however, there is information available about their roles in locomotion. Further, although locomotor functions have been attributed to these populations, it is expected that not all neurons in any class participate in locomotion, but they may contribute to pathways involved in behavioral responses to stimuli described above.

### 6.1. V0 INs

The V0 INs are commissural INs defined by the expression of the transcription factor Dbx1 [144,145]. They are subdivided into dorsal (V0_D_) and ventral (V0_V_) populations based on the absence and presence of Evx1, respectively. The V0_V_ neurons are mainly excitatory and the V0_D_ INs are inhibitory, distinguished by presence of Dbx1 in the absence of Evx1 or the co-expression of Dbx1 and Pax2 [146]. The majority of V0_D_ neurons are glycinergic and about half are GABAergic [146,147]. Medially located V0_D_ neurons receive dense input from vGluT1-expressing terminals, suggesting that these neurons receive monosynaptic input from primary afferents [147]. The V0 INs are essential for left-right alternation during locomotion [145]. In contrast to the polysynaptic inhibition from V0_V_ INs, inhibitory V0_D_ INs form monosynaptic contacts with contralateral motor pools and are directly involved in alternation at low locomotor speeds [146,148], corresponding to walking gait in quadruped animals [149,150,151]. Although a speed-dependent change in commissural IN population involved has been established, the basis of that switch remains unknown and it is likely that proprioceptive input plays a role.

### 6.2. V1 INs

The V1 INs, defined by the expression of Engrailed 1 (En1), are exclusively ipsilaterally projecting inhibitory INs [152]. These neurons are in laminae VII and IX, predominantly glycinergic, and include some of the most well studied populations of inhibitory INs, the Renshaw cells and the Ia inhibitory INs [153].

Renshaw cells make up <10% of the V1 population [153]. They are glycinergic neurons which receive input from axon collaterals of motor neurons and mediate recurrent inhibition [154]. In addition to motor neurons, Renshaw cells inhibit other Renshaw cells, Ia inhibitory INs, and spinocerebellar neurons [155]. Although Renshaw cells receive direct proprioceptive input early in development, this is lost by adulthood [156]. Renshaw cells express both calbindin and PV in mouse [153]. They have also been recently shown to be identified by Chrna2 [157]. Renshaw cells have been hypothesized to function to regulate motor neuron gain [158]. Genetic silencing of Renshaw cells does not impact gait or coordination of locomotion [157]; however, it is possible that there is developmental compensation.

Ia inhibitory INs express PV but not calbindin and receive input from proprioceptive Ia afferents and input from Renshaw cells [153,159]. Ia inhibitory INs make synapses onto antagonist motor neurons to mediate reciprocal inhibition [160,161]. In addition to V1 neurons, a subpopulation of V2b INs (below) also constitute Ia inhibitory INs [162].

Approximately 75% of the V1 INs are not Renshaw cells or Ia inhibitory INs [153]. At least some of the remaining neurons are thought to contribute to flexor-extensor alternation during locomotion. Genetic ablation or silencing of V1 INs does not disrupt coordination but rather decreases the locomotor frequency [162,163] and increases the duration of flexor activation [164].

### 6.3. V2b INs

Similar to the V1 INs, V2b INs are inhibitory, ipsilaterally projecting INs located primarily in lamina VII [165]. A small proportion of V2b INs are Ia inhibitory INs [162]. V2b INs preferentially inhibit extensor motor neurons [164]. Genetic removal of V2b INs in in vitro spinal cords does not result in a locomotor change on its own [162]. However, if the spinal cord is hemisected at the midline, flexor and extensor motor activity synchronizes in the V2b mutants, where it is alternating in wildtype littermates [162]. Additionally, the genetic removal of both V1 and V2b populations results in flexor-extensor synchronization [162], suggesting that constituents of these populations cooperate to secure flexor-extensor alternation [166].

### 6.4. dI6 INs

dI6 INs are identified by the transcription factors Wt1 and/or Dmrt3 [167,168,169]. These are mainly inhibitory commissural INs located in laminae VII and VIII [168,169,170]. dI6 INs are rhythmically active during locomotion [169,171]. The axons of dI6 neurons innervate motor neurons and INs on the contralateral side of the cord [169,170] and silencing leads to deficits in left-right coordination [167,169]. It is currently unknown if the dI6 populations are specific to locomotion at particular speed or gaits. Since Dmrt3 and Wt1 expression can distinguish three populations of inhibitory neurons, it is possible that each has discrete projection targets and specialized function.

## 7. Conclusions and Outlook

The current description of INs across the dorsal and ventral horns has become increasingly complex as functionally distinct populations are identified and interrogated. The populations described above are unlikely to constitute all spinal inhibitory INs, as there are other inhibitory markers that define candidate populations that have been shown to be distinct but have not been studied in detail to date [8,19,20,172,173]. Large scale sequencing studies provide additional detail which may be necessary in order to discern differential patterns of gene expression amongst functionally related and/or overlapping populations [19,20]. Such data have identified at least 18 molecularly distinct groups of inhibitory INs that are not necessarily reflected in the populations described as they are grouped in the current review [8,19,20]. On the other hand, in some cases, the populations are broad and may represent several classes of INs, requiring intersectional strategies to dissect more discrete functional roles [174]. This is particularly the case for populations identified by markers that include excitatory populations [18,109,112,121,126].

The true test of population identity should be function. Genetic access to molecularly distinct populations has served to complement previous classification schemes and enable targeted manipulations. Even so, this approach may not capture the full extent of effect from a given population. Further, the idea that molecularly defined neurons will delineate functional pathways, although attractive, has been more recently called into question. Instead, synaptic connectivity and population coding considered together with cell-specific markers is more likely to be required to define functional circuits [10,57,139]. Future functional studies taking these features into account will be challenging but highly valuable.

Inhibitory microcircuits involved in afferent gating represent robust and highly conserved mechanisms employed by the central nervous system to regulate neurotransmission. Dissecting spinal circuits will be one key step towards unraveling the intricacies of neural circuit organization and signaling. Reflexive responses and locomotion are highly stereotypic behaviors that are amenable to observation, measurement, and association with individual spinal IN populations, and more complex responses are underexplored to date. The cumulative findings from targeted genetic manipulations offer several candidate populations for novel therapeutic interventions and functional recovery after injury [123,175], however, several aspects surrounding these strategies impede effective clinical translation. For example, the identity and role of supraspinal structures and additional neuromodulatory or peptidergic signaling pathways are not clear despite anatomical and physiological evidence of their recruitment. Additionally, genes of interest are often expressed in multiple populations, in the spinal cord and other regions of the CNS, and we lack understanding of how these common neurons are affected by selective manipulations. Recent exploration of brainstem circuits has identified integrative nodes that play a role in motor initiation and will be critical to ascertain a holistic understanding of sensorimotor pathways, particularly given that these same neurons are likely to integrate signals from these higher order centers [176,177,178]. Enhancement of behavioral assays to detect subtleties and behavioral changes in more natural scenarios, perhaps by implementing machine learning and/or kinematic analysis [179] will greatly benefit these efforts. Given our current understanding of spinal sensorimotor signaling, future research endeavors aimed at enhanced genetic selectivity based off of next generation sequencing and improved discrimination of subtle behaviors will allow for further probing that will be necessary to understand the mechanistic underpinnings for the remarkable repertoire of sensory-evoked behaviors.

## References

[1] Wall P.D., Dubner R. (1972). Somatosensory pathways. Annu. Rev. Physiol..

[2] Willis W.D. (2007). The somatosensory system, with emphasis on structures important for pain. Brain Res. Rev..

[3] Todd A.J. (2010). Neuronal circuitry for pain processing in the dorsal horn. Nat. Rev. Neurosci..

[4] Lu Y., Perl E.R. (2005). Modular organization of excitatory circuits between neurons of the spinal superficial dorsal horn (laminae I and II). J. Neurosci..

[5] Jankowska E., Maxwell D.J., Bannatyne B.A. (2007). On coupling and decoupling of spinal interneuronal networks. Arch. Ital. Biol..

[6] Calvino B., Grilo R.M. (2006). Central pain control. Joint Bone Spine.

[7] Levine A.J., Lewallen K.A., Pfaff S.L. (2012). Spatial organization of cortical and spinal neurons controlling motor behavior. Curr. Opin. Neurobiol..

[8] Abraira V.E., Kuehn E.D., Chirila A.M., Springel M.W., Toliver A.A., Zimmerman A.L., Orefice L.L., Boyle K.A., Bai L., Song B.J. (2017). The Cellular and Synaptic Architecture of the Mechanosensory Dorsal Horn. Cell.

[9] Peirs C., Dallel R., Todd A.J. (2020). Recent advances in our understanding of the organization of dorsal horn neuron populations and their contribution to cutaneous mechanical allodynia. J. Neural. Transm..

[10] Prescott S.A., Ma Q., De Koninck Y. (2014). Normal and abnormal coding of somatosensory stimuli causing pain. Nat. Neurosci..

[11] Melzack R., Wall P.D. (1965). Pain mechanisms: A new theory. Science.

[12] Bardoni R., Takazawa T., Tong C.K., Choudhury P., Scherrer G., Macdermott A.B. (2013). Pre- and postsynaptic inhibitory control in the spinal cord dorsal horn. Ann. N. Y. Acad. Sci..

[13] Hughes D.I., Todd A.J. (2020). Central Nervous System Targets: Inhibitory Interneurons in the Spinal Cord. Neurotherapeutics.

[14] Guo D., Hu J. (2014). Spinal presynaptic inhibition in pain control. Neuroscience.

[15] Eccles J.C., Schmidt R.F., Willis W.D. (1962). Presynaptic inhibition of the spinal monosynaptic reflex pathway. J. Physiol..

[16] Grudt T.J., Perl E.R. (2002). Correlations between neuronal morphology and electrophysiological features in the rodent superficial dorsal horn. J. Physiol..

[17] Goulding M., Bourane S., Garcia-Campmany L., Dalet A., Koch S. (2014). Inhibition downunder: An update from the spinal cord. Curr. Opin. Neurobiol..

[18] Boyle K.A., Gutierrez-Mecinas M., Polgar E., Mooney N., O’Connor E., Furuta T., Watanabe M., Todd A.J. (2017). A quantitative study of neurochemically defined populations of inhibitory interneurons in the superficial dorsal horn of the mouse spinal cord. Neuroscience.

[19] Sathyamurthy A., Johnson K.R., Matson K.J.E., Dobrott C.I., Li L., Ryba A.R., Bergman T.B., Kelly M.C., Kelley M.W., Levine A.J. (2018). Massively Parallel Single Nucleus Transcriptional Profiling Defines Spinal Cord Neurons and Their Activity during Behavior. Cell Rep..

[20] Haring M., Zeisel A., Hochgerner H., Rinwa P., Jakobsson J.E.T., Lonnerberg P., La Manno G., Sharma N., Borgius L., Kiehn O. (2018). Neuronal atlas of the dorsal horn defines its architecture and links sensory input to transcriptional cell types. Nat. Neurosci..

[21] Abraira V.E., Ginty D.D. (2013). The sensory neurons of touch. Neuron.

[22] Lai H.C., Seal R.P., Johnson J.E. (2016). Making sense out of spinal cord somatosensory development. Development.

[23] Horch K.W., Tuckett R.P., Burgess P.R. (1977). A key to the classification of cutaneous mechanoreceptors. J. Investig. Dermatol..

[24] Harper A.A., Lawson S.N. (1985). Conduction velocity is related to morphological cell type in rat dorsal root ganglion neurones. J. Physiol..

[25] Leem J.W., Willis W.D., Chung J.M. (1993). Cutaneous sensory receptors in the rat foot. J. Neurophysiol..

[26] Light A.R., Perl E.R. (1979). Spinal termination of functionally identified primary afferent neurons with slowly conducting myelinated fibers. J. Comp. Neurol..

[27] Edgley S.A., Jankowska E. (1987). Field potentials generated by group II muscle afferents in the middle lumbar segments of the cat spinal cord. J. Physiol..

[28] Mense S., Craig A.D. (1988). Spinal and supraspinal terminations of primary afferent fibers from the gastrocnemius-soleus muscle in the cat. Neuroscience.

[29] Neumann S., Braz J.M., Skinner K., Llewellyn-Smith I.J., Basbaum A.I. (2008). Innocuous, not noxious, input activates PKCgamma interneurons of the spinal dorsal horn via myelinated afferent fibers. J. Neurosci..

[30] Sugiura Y., Lee C.L., Perl E.R. (1986). Central projections of identified, unmyelinated (C) afferent fibers innervating mammalian skin. Science.

[31] Basbaum A.I., Bautista D.M., Scherrer G., Julius D. (2009). Cellular and molecular mechanisms of pain. Cell.

[32] Braz J., Solorzano C., Wang X., Basbaum A.I. (2014). Transmitting pain and itch messages: A contemporary view of the spinal cord circuits that generate gate control. Neuron.

[33] Woolf C.J., Ma Q. (2007). Nociceptors—Noxious stimulus detectors. Neuron.

[34] Cavanaugh D.J., Lee H., Lo L., Shields S.D., Zylka M.J., Basbaum A.I., Anderson D.J. (2009). Distinct subsets of unmyelinated primary sensory fibers mediate behavioral responses to noxious thermal and mechanical stimuli. Proc. Natl. Acad. Sci. USA.

[35] Zhang J., Cavanaugh D.J., Nemenov M.I., Basbaum A.I. (2013). The modality-specific contribution of peptidergic and non-peptidergic nociceptors is manifest at the level of dorsal horn nociresponsive neurons. J. Physiol..

[36] Julius D. (2013). TRP channels and pain. Annu. Rev. Cell Dev. Biol..

[37] Seal R.P., Wang X., Guan Y., Raja S.N., Woodbury C.J., Basbaum A.I., Edwards R.H. (2009). Injury-induced mechanical hypersensitivity requires C-low threshold mechanoreceptors. Nature.

[38] Larsson M., Broman J. (2019). Synaptic Organization of VGLUT3 Expressing Low-Threshold Mechanosensitive C Fiber Terminals in the Rodent Spinal Cord. eNeuro.

[39] Liu Y., Ma Q. (2011). Generation of somatic sensory neuron diversity and implications on sensory coding. Curr. Opin. Neurobiol..

[40] Usoskin D., Furlan A., Islam S., Abdo H., Lonnerberg P., Lou D., Hjerling-Leffler J., Haeggstrom J., Kharchenko O., Kharchenko P.V. (2015). Unbiased classification of sensory neuron types by large-scale single-cell RNA sequencing. Nat. Neurosci..

[41] Ranade S.S., Woo S.H., Dubin A.E., Moshourab R.A., Wetzel C., Petrus M., Mathur J., Begay V., Coste B., Mainquist J. (2014). Piezo2 is the major transducer of mechanical forces for touch sensation in mice. Nature.

[42] Hill R.Z., Bautista D.M. (2020). Getting in Touch with Mechanical Pain Mechanisms. Trends Neurosci..

[43] Betley J.N., Wright C.V., Kawaguchi Y., Erdelyi F., Szabo G., Jessell T.M., Kaltschmidt J.A. (2009). Stringent specificity in the construction of a GABAergic presynaptic inhibitory circuit. Cell.

[44] Levine A.J., Hinckley C.A., Hilde K.L., Driscoll S.P., Poon T.H., Montgomery J.M., Pfaff S.L. (2014). Identification of a cellular node for motor control pathways. Nat. Neurosci..

[45] De Nooij J.C., Doobar S., Jessell T.M. (2013). Etv1 inactivation reveals proprioceptor subclasses that reflect the level of NT3 expression in muscle targets. Neuron.

[46] Wu D., Schieren I., Qian Y., Zhang C., Jessell T.M., de Nooij J.C. (2019). A Role for Sensory end Organ-Derived Signals in Regulating Muscle Spindle Proprioceptor Phenotype. J. Neurosci..

[47] Wu H., Petitpre C., Fontanet P., Sharma A., Bellardita C., Quadros R.M., Jannig P.R., Wang Y., Heimel J.A., Cheung K.K.Y. (2021). Distinct subtypes of proprioceptive dorsal root ganglion neurons regulate adaptive proprioception in mice. Nat. Commun..

[48] Koch S.C., Acton D., Goulding M. (2018). Spinal Circuits for Touch, Pain, and Itch. Annu. Rev. Physiol..

[49] Gatto G., Smith K.M., Ross S.E., Goulding M. (2019). Neuronal diversity in the somatosensory system: Bridging the gap between cell type and function. Curr. Opin. Neurobiol..

[50] Bourane S., Grossmann K.S., Britz O., Dalet A., Del Barrio M.G., Stam F.J., Garcia-Campmany L., Koch S., Goulding M. (2015). Identification of a spinal circuit for light touch and fine motor control. Cell.

[51] Mu D., Deng J., Liu K.F., Wu Z.Y., Shi Y.F., Guo W.M., Mao Q.Q., Liu X.J., Li H., Sun Y.G. (2017). A central neural circuit for itch sensation. Science.

[52] Pan H., Fatima M., Li A., Lee H., Cai W., Horwitz L., Hor C.C., Zaher N., Cin M., Slade H. (2019). Identification of a Spinal Circuit for Mechanical and Persistent Spontaneous Itch. Neuron.

[53] Petitjean H., Bourojeni F.B., Tsao D., Davidova A., Sotocinal S.G., Mogil J.S., Kania A., Sharif-Naeini R. (2019). Recruitment of Spinoparabrachial Neurons by Dorsal Horn Calretinin Neurons. Cell Rep..

[54] Artola A., Voisin D., Dallel R. (2020). PKCgamma interneurons, a gateway to pathological pain in the dorsal horn. J. Neural Transm..

[55] Cameron D., Polgar E., Gutierrez-Mecinas M., Gomez-Lima M., Watanabe M., Todd A.J. (2015). The organisation of spinoparabrachial neurons in the mouse. Pain.

[56] Hachisuka J., Koerber H.R., Ross S.E. (2020). Selective-cold output through a distinct subset of lamina I spinoparabrachial neurons. Pain.

[57] Ma Q. (2010). Labeled lines meet and talk: Population coding of somatic sensations. J. Clin. Investig..

[58] Prescott S.A., Ratte S. (2012). Pain processing by spinal microcircuits: Afferent combinatorics. Curr. Opin. Neurobiol..

[59] Bui T.V., Stifani N., Panek I., Farah C. (2015). Genetically identified spinal interneurons integrating tactile afferents for motor control. J. Neurophysiol..

[60] Polgar E., Thomson S., Maxwell D.J., Al-Khater K., Todd A.J. (2007). A population of large neurons in laminae III and IV of the rat spinal cord that have long dorsal dendrites and lack the neurokinin 1 receptor. Eur. J. Neurosci..

[61] Fernandes E.C., Santos I.C., Kokai E., Luz L.L., Szucs P., Safronov B.V. (2018). Low- and high-threshold primary afferent inputs to spinal lamina III antenna-type neurons. Pain.

[62] Wercberger R., Basbaum A.I. (2019). Spinal cord projection neurons: A superficial, and also deep, analysis. Curr. Opin. Physiol..

[63] Hantman A.W., Jessell T.M. (2010). Clarke’s column neurons as the focus of a corticospinal corollary circuit. Nat. Neurosci..

[64] Azim E., Seki K. (2019). Gain control in the sensorimotor system. Curr. Opin. Physiol..

[65] Kiehn O. (2016). Decoding the organization of spinal circuits that control locomotion. Nat. Rev. Neurosci..

[66] Gosgnach S., Bikoff J.B., Dougherty K.J., El Manira A., Lanuza G.M., Zhang Y. (2017). Delineating the Diversity of Spinal Interneurons in Locomotor Circuits. J. Neurosci..

[67] Cote M.P., Murray L.M., Knikou M. (2018). Spinal Control of Locomotion: Individual Neurons, Their Circuits and Functions. Front. Physiol..

[68] Dougherty K.J., Ha N.T. (2019). The rhythm section: An update on spinal interneurons setting the beat for mammalian locomotion. Curr. Opin. Physiol..

[69] Goulding M. (2009). Circuits controlling vertebrate locomotion: Moving in a new direction. Nat. Rev. Neurosci..

[70] Frigon A. (2012). Central pattern generators of the mammalian spinal cord. Neuroscientist.

[71] Akay T. (2020). Sensory Feedback Control of Locomotor Pattern Generation in Cats and Mice. Neuroscience.

[72] Rossignol S., Dubuc R., Gossard J.P. (2006). Dynamic sensorimotor interactions in locomotion. Physiol. Rev..

[73] Kriellaars D.J., Brownstone R.M., Noga B.R., Jordan L.M. (1994). Mechanical entrainment of fictive locomotion in the decerebrate cat. J. Neurophysiol..

[74] Forssberg H., Grillner S., Rossignol S. (1977). Phasic gain control of reflexes from the dorsum of the paw during spinal locomotion. Brain Res..

[75] Forssberg H. (1979). Stumbling corrective reaction: A phase-dependent compensatory reaction during locomotion. J. Neurophysiol..

[76] Zeilhofer H.U., Wildner H., Yevenes G.E. (2012). Fast synaptic inhibition in spinal sensory processing and pain control. Physiol. Rev..

[77] Price T.J., Prescott S.A. (2015). Inhibitory regulation of the pain gate and how its failure causes pathological pain. Pain.

[78] Todd A.J., Sullivan A.C. (1990). Light microscope study of the coexistence of GABA-like and glycine-like immunoreactivities in the spinal cord of the rat. J. Comp. Neurol..

[79] Dougherty K.J., Sawchuk M.A., Hochman S. (2009). Phenotypic diversity and expression of GABAergic inhibitory interneurons during postnatal development in lumbar spinal cord of glutamic acid decarboxylase 67-green fluorescent protein mice. Neuroscience.

[80] Mackie M., Hughes D.I., Maxwell D.J., Tillakaratne N.J., Todd A.J. (2003). Distribution and colocalisation of glutamate decarboxylase isoforms in the rat spinal cord. Neuroscience.

[81] Foster E., Wildner H., Tudeau L., Haueter S., Ralvenius W.T., Jegen M., Johannssen H., Hosli L., Haenraets K., Ghanem A. (2015). Targeted ablation, silencing, and activation establish glycinergic dorsal horn neurons as key components of a spinal gate for pain and itch. Neuron.

[82] Inquimbert P., Rodeau J.L., Schlichter R. (2007). Differential contribution of GABAergic and glycinergic components to inhibitory synaptic transmission in lamina II and laminae III-IV of the young rat spinal cord. Eur. J. Neurosci..

[83] Takazawa T., MacDermott A.B. (2010). Glycinergic and GABAergic tonic inhibition fine tune inhibitory control in regionally distinct subpopulations of dorsal horn neurons. J. Physiol..

[84] Takazawa T., Choudhury P., Tong C.K., Conway C.M., Scherrer G., Flood P.D., Mukai J., MacDermott A.B. (2017). Inhibition Mediated by Glycinergic and GABAergic Receptors on Excitatory Neurons in Mouse Superficial Dorsal Horn Is Location-Specific but Modified by Inflammation. J. Neurosci..

[85] Levy R.A. (1977). The role of GABA in primary afferent depolarization. Prog. Neurobiol..

[86] Rudomin P., Schmidt R.F. (1999). Presynaptic inhibition in the vertebrate spinal cord revisited. Exp. Brain Res..

[87] Hughes D.I., Mackie M., Nagy G.G., Riddell J.S., Maxwell D.J., Szabo G., Erdelyi F., Veress G., Szucs P., Antal M. (2005). P boutons in lamina IX of the rodent spinal cord express high levels of glutamic acid decarboxylase-65 and originate from cells in deep medial dorsal horn. Proc. Natl. Acad. Sci. USA.

[88] Mende M., Fletcher E.V., Belluardo J.L., Pierce J.P., Bommareddy P.K., Weinrich J.A., Kabir Z.D., Schierberl K.C., Pagiazitis J.G., Mendelsohn A.I. (2016). Sensory-Derived Glutamate Regulates Presynaptic Inhibitory Terminals in Mouse Spinal Cord. Neuron.

[89] Rudomin P. (2009). In search of lost presynaptic inhibition. Exp. Brain Res..

[90] Lee K.J., Jessell T.M. (1999). The specification of dorsal cell fates in the vertebrate central nervous system. Annu. Rev. Neurosci..

[91] Brohl D., Strehle M., Wende H., Hori K., Bormuth I., Nave K.A., Muller T., Birchmeier C. (2008). A transcriptional network coordinately determines transmitter and peptidergic fate in the dorsal spinal cord. Dev. Biol..

[92] Glasgow S.M., Henke R.M., Macdonald R.J., Wright C.V., Johnson J.E. (2005). Ptf1a determines GABAergic over glutamatergic neuronal cell fate in the spinal cord dorsal horn. Development.

[93] Pillai A., Mansouri A., Behringer R., Westphal H., Goulding M. (2007). Lhx1 and Lhx5 maintain the inhibitory-neurotransmitter status of interneurons in the dorsal spinal cord. Development.

[94] Huang M., Huang T., Xiang Y., Xie Z., Chen Y., Yan R., Xu J., Cheng L. (2008). Ptf1a, Lbx1 and Pax2 coordinate glycinergic and peptidergic transmitter phenotypes in dorsal spinal inhibitory neurons. Dev. Biol..

[95] Larsson M. (2017). Pax2 is persistently expressed by GABAergic neurons throughout the adult rat dorsal horn. Neurosci. Lett..

[96] Polgar E., Durrieux C., Hughes D.I., Todd A.J. (2013). A quantitative study of inhibitory interneurons in laminae I-III of the mouse spinal dorsal horn. PLoS ONE.

[97] Benito-Gonzalez A., Alvarez F.J. (2012). Renshaw cells and Ia inhibitory interneurons are generated at different times from p1 progenitors and differentiate shortly after exiting the cell cycle. J. Neurosci..

[98] Browne T.J., Hughes D.I., Dayas C.V., Callister R.J., Graham B.A. (2020). Projection Neuron Axon Collaterals in the Dorsal Horn: Placing a New Player in Spinal Cord Pain Processing. Front. Physiol..

[99] Yamamoto T., Carr P.A., Baimbridge K.G., Nagy J.I. (1989). Parvalbumin- and calbindin D28k-immunoreactive neurons in the superficial layers of the spinal cord dorsal horn of rat. Brain Res. Bull..

[100] Tiong S.Y., Polgar E., van Kralingen J.C., Watanabe M., Todd A.J. (2011). Galanin-immunoreactivity identifies a distinct population of inhibitory interneurons in laminae I-III of the rat spinal cord. Mol. Pain.

[101] Hughes D.I., Sikander S., Kinnon C.M., Boyle K.A., Watanabe M., Callister R.J., Graham B.A. (2012). Morphological, neurochemical and electrophysiological features of parvalbumin-expressing cells: A likely source of axo-axonic inputs in the mouse spinal dorsal horn. J. Physiol..

[102] Petitjean H., Pawlowski S.A., Fraine S.L., Sharif B., Hamad D., Fatima T., Berg J., Brown C.M., Jan L.Y., Ribeiro-da-Silva A. (2015). Dorsal Horn Parvalbumin Neurons Are Gate-Keepers of Touch-Evoked Pain after Nerve Injury. Cell Rep..

[103] Laing I., Todd A.J., Heizmann C.W., Schmidt H.H. (1994). Subpopulations of GABAergic neurons in laminae I-III of rat spinal dorsal horn defined by coexistence with classical transmitters, peptides, nitric oxide synthase or parvalbumin. Neuroscience.

[104] Heinke B., Ruscheweyh R., Forsthuber L., Wunderbaldinger G., Sandkuhler J. (2004). Physiological, neurochemical and morphological properties of a subgroup of GABAergic spinal lamina II neurones identified by expression of green fluorescent protein in mice. J. Physiol..

[105] Boyle K.A., Gradwell M.A., Yasaka T., Dickie A.C., Polgar E., Ganley R.P., Orr D.P.H., Watanabe M., Abraira V.E., Kuehn E.D. (2019). Defining a Spinal Microcircuit that Gates Myelinated Afferent Input: Implications for Tactile Allodynia. Cell Rep..

[106] Tamamaki N., Yanagawa Y., Tomioka R., Miyazaki J., Obata K., Kaneko T. (2003). Green fluorescent protein expression and colocalization with calretinin, parvalbumin, and somatostatin in the GAD67-GFP knock-in mouse. J. Comp. Neurol..

[107] Todd A.J. (1996). GABA and glycine in synaptic glomeruli of the rat spinal dorsal horn. Eur. J. Neurosci..

[108] Gradwell M.A., Boyle K.A., Callister R.J., Hughes D.I., Graham B.A. (2017). Heteromeric alpha/beta glycine receptors regulate excitability in parvalbumin-expressing dorsal horn neurons through phasic and tonic glycinergic inhibition. J. Physiol..

[109] Sardella T.C., Polgar E., Garzillo F., Furuta T., Kaneko T., Watanabe M., Todd A.J. (2011). Dynorphin is expressed primarily by GABAergic neurons that contain galanin in the rat dorsal horn. Mol. Pain.

[110] Kardon A.P., Polgar E., Hachisuka J., Snyder L.M., Cameron D., Savage S., Cai X., Karnup S., Fan C.R., Hemenway G.M. (2014). Dynorphin acts as a neuromodulator to inhibit itch in the dorsal horn of the spinal cord. Neuron.

[111] Simmons D.R., Spike R.C., Todd A.J. (1995). Galanin is contained in GABAergic neurons in the rat spinal dorsal horn. Neurosci. Lett..

[112] Huang J., Polgar E., Solinski H.J., Mishra S.K., Tseng P.Y., Iwagaki N., Boyle K.A., Dickie A.C., Kriegbaum M.C., Wildner H. (2018). Circuit dissection of the role of somatostatin in itch and pain. Nat. Neurosci..

[113] Duan B., Cheng L., Bourane S., Britz O., Padilla C., Garcia-Campmany L., Krashes M., Knowlton W., Velasquez T., Ren X. (2014). Identification of spinal circuits transmitting and gating mechanical pain. Cell.

[114] Brewer C.L., Styczynski L.M., Serafin E.K., Baccei M.L. (2020). Postnatal maturation of spinal dynorphin circuits and their role in somatosensation. Pain.

[115] Liu M.Z., Chen X.J., Liang T.Y., Li Q., Wang M., Zhang X.Y., Li Y.Z., Sun Q., Sun Y.G. (2019). Synaptic control of spinal GRPR(+) neurons by local and long-range inhibitory inputs. Proc. Natl. Acad. Sci. USA.

[116] Albisetti G.W., Pagani M., Platonova E., Hosli L., Johannssen H.C., Fritschy J.M., Wildner H., Zeilhofer H.U. (2019). Dorsal Horn Gastrin-Releasing Peptide Expressing Neurons Transmit Spinal Itch But Not Pain Signals. J. Neurosci..

[117] Bernardi P.S., Valtschanoff J.G., Weinberg R.J., Schmidt H.H., Rustioni A. (1995). Synaptic interactions between primary afferent terminals and GABA and nitric oxide-synthesizing neurons in superficial laminae of the rat spinal cord. J. Neurosci..

[118] Polgar E., Sardella T.C.P., Tiong S.Y.X., Locke S., Watanabe M., Todd A.J. (2013). Functional differences between neurochemically defined populations of inhibitory interneurons in the rat spinal dorsal horn. Pain.

[119] Puskar Z., Polgar E., Todd A.J. (2001). A population of large lamina I projection neurons with selective inhibitory input in rat spinal cord. Neuroscience.

[120] Sardella T.C., Polgar E., Watanabe M., Todd A.J. (2011). A quantitative study of neuronal nitric oxide synthase expression in laminae I-III of the rat spinal dorsal horn. Neuroscience.

[121] Iwagaki N., Ganley R.P., Dickie A.C., Polgar E., Hughes D.I., Del Rio P., Revina Y., Watanabe M., Todd A.J., Riddell J.S. (2016). A combined electrophysiological and morphological study of neuropeptide Y-expressing inhibitory interneurons in the spinal dorsal horn of the mouse. Pain.

[122] Bourane S., Duan B., Koch S.C., Dalet A., Britz O., Garcia-Campmany L., Kim E., Cheng L., Ghosh A., Ma Q. (2015). Gate control of mechanical itch by a subpopulation of spinal cord interneurons. Science.

[123] Tashima R., Koga K., Yoshikawa Y., Sekine M., Watanabe M., Tozaki-Saitoh H., Furue H., Yasaka T., Tsuda M. (2021). A subset of spinal dorsal horn interneurons crucial for gating touch-evoked pain-like behavior. Proc. Natl. Acad. Sci. USA.

[124] Acton D., Ren X., Di Costanzo S., Dalet A., Bourane S., Bertocchi I., Eva C., Goulding M. (2019). Spinal Neuropeptide Y1 Receptor-Expressing Neurons Form an Essential Excitatory Pathway for Mechanical Itch. Cell Rep..

[125] Polgar E., Shehab S.A., Watt C., Todd A.J. (1999). GABAergic neurons that contain neuropeptide Y selectively target cells with the neurokinin 1 receptor in laminae III and IV of the rat spinal cord. J. Neurosci..

[126] Smith K.M., Boyle K.A., Madden J.F., Dickinson S.A., Jobling P., Callister R.J., Hughes D.I., Graham B.A. (2015). Functional heterogeneity of calretinin-expressing neurons in the mouse superficial dorsal horn: Implications for spinal pain processing. J. Physiol..

[127] Ren K., Ruda M.A., Jacobowitz D.M. (1993). Immunohistochemical localization of calretinin in the dorsal root ganglion and spinal cord of the rat. Brain Res. Bull..

[128] Gutierrez-Mecinas M., Davis O., Polgar E., Shahzad M., Navarro-Batista K., Furuta T., Watanabe M., Hughes D.I., Todd A.J. (2019). Expression of Calretinin Among Different Neurochemical Classes of Interneuron in the Superficial Dorsal Horn of the Mouse Spinal Cord. Neuroscience.

[129] Smith K.M., Boyle K.A., Mustapa M., Jobling P., Callister R.J., Hughes D.I., Graham B.A. (2016). Distinct forms of synaptic inhibition and neuromodulation regulate calretinin-positive neuron excitability in the spinal cord dorsal horn. Neuroscience.

[130] Prochazka A. (1989). Sensorimotor gain control: A basic strategy of motor systems?. Prog. Neurobiol..

[131] Panek I., Bui T., Wright A.T., Brownstone R.M. (2014). Cutaneous afferent regulation of motor function. Acta Neurobiol. Exp..

[132] Cui L., Miao X., Liang L., Abdus-Saboor I., Olson W., Fleming M.S., Ma M., Tao Y.X., Luo W. (2016). Identification of Early RET+ Deep Dorsal Spinal Cord Interneurons in Gating Pain. Neuron.

[133] Koch S.C., Del Barrio M.G., Dalet A., Gatto G., Gunther T., Zhang J., Seidler B., Saur D., Schule R., Goulding M. (2017). RORbeta Spinal Interneurons Gate Sensory Transmission during Locomotion to Secure a Fluid Walking Gait. Neuron.

[134] Andre E., Conquet F., Steinmayr M., Stratton S.C., Porciatti V., Becker-Andre M. (1998). Disruption of retinoid-related orphan receptor beta changes circadian behavior, causes retinal degeneration and leads to vacillans phenotype in mice. EMBO J..

[135] Hilde K.L., Levine A.J., Hinckley C.A., Hayashi M., Montgomery J.M., Gullo M., Driscoll S.P., Grosschedl R., Kohwi Y., Kohwi-Shigematsu T. (2016). Satb2 Is Required for the Development of a Spinal Exteroceptive Microcircuit that Modulates Limb Position. Neuron.

[136] Brownstone R.M., Bui T.V. (2010). Spinal interneurons providing input to the final common path during locomotion. Prog. Brain Res..

[137] Akay T., Tourtellotte W.G., Arber S., Jessell T.M. (2014). Degradation of mouse locomotor pattern in the absence of proprioceptive sensory feedback. Proc. Natl. Acad. Sci. USA.

[138] Paixao S., Loschek L., Gaitanos L., Alcala Morales P., Goulding M., Klein R. (2019). Identification of Spinal Neurons Contributing to the Dorsal Column Projection Mediating Fine Touch and Corrective Motor Movements. Neuron.

[139] Gatto G., Bourane S., Ren X., Di Costanzo S., Fenton P.K., Halder P., Seal R.P., Goulding M.D. (2021). A Functional Topographic Map for Spinal Sensorimotor Reflexes. Neuron.

[140] McCrea D.A., Shefchyk S.J., Stephens M.J., Pearson K.G. (1995). Disynaptic group I excitation of synergist ankle extensor motoneurones during fictive locomotion in the cat. J. Physiol..

[141] Buschges A., Manira A.E. (1998). Sensory pathways and their modulation in the control of locomotion. Curr. Opin. Neurobiol..

[142] Quevedo J., Stecina K., McCrea D.A. (2005). Intracellular analysis of reflex pathways underlying the stumbling corrective reaction during fictive locomotion in the cat. J. Neurophysiol..

[143] Jessell T.M. (2000). Neuronal specification in the spinal cord: Inductive signals and transcriptional codes. Nat. Rev. Genet..

[144] Pierani A., Moran-Rivard L., Sunshine M.J., Littman D.R., Goulding M., Jessell T.M. (2001). Control of interneuron fate in the developing spinal cord by the progenitor homeodomain protein Dbx1. Neuron.

[145] Lanuza G.M., Gosgnach S., Pierani A., Jessell T.M., Goulding M. (2004). Genetic identification of spinal interneurons that coordinate left-right locomotor activity necessary for walking movements. Neuron.

[146] Talpalar A.E., Bouvier J., Borgius L., Fortin G., Pierani A., Kiehn O. (2013). Dual-mode operation of neuronal networks involved in left-right alternation. Nature.

[147] Griener A., Zhang W., Kao H., Wagner C., Gosgnach S. (2015). Probing diversity within subpopulations of locomotor-related V0 interneurons. Dev. Neurobiol..

[148] Shevtsova N.A., Talpalar A.E., Markin S.N., Harris-Warrick R.M., Kiehn O., Rybak I.A. (2015). Organization of left-right coordination of neuronal activity in the mammalian spinal cord: Insights from computational modelling. J. Physiol..

[149] Bellardita C., Kiehn O. (2015). Phenotypic characterization of speed-associated gait changes in mice reveals modular organization of locomotor networks. Curr. Biol..

[150] Danner S.M., Wilshin S.D., Shevtsova N.A., Rybak I.A. (2016). Central control of interlimb coordination and speed-dependent gait expression in quadrupeds. J. Physiol..

[151] Danner S.M., Shevtsova N.A., Frigon A., Rybak I.A. (2017). Computational modeling of spinal circuits controlling limb coordination and gaits in quadrupeds. eLife.

[152] Sapir T., Geiman E.J., Wang Z., Velasquez T., Mitsui S., Yoshihara Y., Frank E., Alvarez F.J., Goulding M. (2004). Pax6 and engrailed 1 regulate two distinct aspects of renshaw cell development. J. Neurosci..

[153] Alvarez F.J., Jonas P.C., Sapir T., Hartley R., Berrocal M.C., Geiman E.J., Todd A.J., Goulding M. (2005). Postnatal phenotype and localization of spinal cord V1 derived interneurons. J. Comp. Neurol..

[154] Eccles J.C., Fatt P., Koketsu K. (1954). Cholinergic and inhibitory synapses in a pathway from motor-axon collaterals to motoneurones. J. Physiol..

[155] Alvarez F.J., Fyffe R.E. (2007). The continuing case for the Renshaw cell. J. Physiol..

[156] Mentis G.Z., Siembab V.C., Zerda R., O’Donovan M.J., Alvarez F.J. (2006). Primary afferent synapses on developing and adult Renshaw cells. J. Neurosci..

[157] Enjin A., Perry S., Hilscher M.M., Nagaraja C., Larhammar M., Gezelius H., Eriksson A., Leao K.E., Kullander K. (2017). Developmental Disruption of Recurrent Inhibitory Feedback Results in Compensatory Adaptation in the Renshaw Cell-Motor Neuron Circuit. J. Neurosci..

[158] Hultborn H., Brownstone R.B., Toth T.I., Gossard J.P. (2004). Key mechanisms for setting the input-output gain across the motoneuron pool. Prog. Brain Res..

[159] Siembab V.C., Smith C.A., Zagoraiou L., Berrocal M.C., Mentis G.Z., Alvarez F.J. (2010). Target selection of proprioceptive and motor axon synapses on neonatal V1-derived Ia inhibitory interneurons and Renshaw cells. J. Comp. Neurol..

[160] Eccles J.C., Fatt P., Landgren S. (1956). Central pathway for direct inhibitory action of impulses in largest afferent nerve fibres to muscle. J. Neurophysiol..

[161] Hultborn H., Illert M., Santini M. (1976). Convergence on interneurones mediating the reciprocal Ia inhibition of motoneurones. III. Effects from supraspinal pathways. Acta Physiol. Scand..

[162] Zhang J., Lanuza G.M., Britz O., Wang Z., Siembab V.C., Zhang Y., Velasquez T., Alvarez F.J., Frank E., Goulding M. (2014). V1 and v2b interneurons secure the alternating flexor-extensor motor activity mice require for limbed locomotion. Neuron.

[163] Gosgnach S., Lanuza G.M., Butt S.J., Saueressig H., Zhang Y., Velasquez T., Riethmacher D., Callaway E.M., Kiehn O., Goulding M. (2006). V1 spinal neurons regulate the speed of vertebrate locomotor outputs. Nature.

[164] Britz O., Zhang J., Grossmann K.S., Dyck J., Kim J.C., Dymecki S., Gosgnach S., Goulding M. (2015). A genetically defined asymmetry underlies the inhibitory control of flexor-extensor locomotor movements. eLife.

[165] Lundfald L., Restrepo C.E., Butt S.J., Peng C.Y., Droho S., Endo T., Zeilhofer H.U., Sharma K., Kiehn O. (2007). Phenotype of V2-derived interneurons and their relationship to the axon guidance molecule EphA4 in the developing mouse spinal cord. Eur. J. Neurosci..

[166] Shevtsova N.A., Rybak I.A. (2016). Organization of flexor-extensor interactions in the mammalian spinal cord: Insights from computational modelling. J. Physiol..

[167] Andersson L.S., Larhammar M., Memic F., Wootz H., Schwochow D., Rubin C.J., Patra K., Arnason T., Wellbring L., Hjalm G. (2012). Mutations in DMRT3 affect locomotion in horses and spinal circuit function in mice. Nature.

[168] Griener A., Zhang W., Kao H., Haque F., Gosgnach S. (2017). Anatomical and electrophysiological characterization of a population of dI6 interneurons in the neonatal mouse spinal cord. Neuroscience.

[169] Haque F., Rancic V., Zhang W., Clugston R., Ballanyi K., Gosgnach S. (2018). WT1-Expressing Interneurons Regulate Left-Right Alternation during Mammalian Locomotor Activity. J. Neurosci..

[170] Perry S., Larhammar M., Vieillard J., Nagaraja C., Hilscher M.M., Tafreshiha A., Rofo F., Caixeta F.V., Kullander K. (2019). Characterization of Dmrt3-Derived Neurons Suggest a Role within Locomotor Circuits. J. Neurosci..

[171] Dyck J., Lanuza G.M., Gosgnach S. (2012). Functional characterization of dI6 interneurons in the neonatal mouse spinal cord. J. Neurophysiol..

[172] Wildner H., Das Gupta R., Brohl D., Heppenstall P.A., Zeilhofer H.U., Birchmeier C. (2013). Genome-wide expression analysis of Ptf1a- and Ascl1-deficient mice reveals new markers for distinct dorsal horn interneuron populations contributing to nociceptive reflex plasticity. J. Neurosci..

[173] Zhang J., Weinrich J.A.P., Russ J.B., Comer J.D., Bommareddy P.K., DiCasoli R.J., Wright C.V.E., Li Y., van Roessel P.J., Kaltschmidt J.A. (2017). A Role for Dystonia-Associated Genes in Spinal GABAergic Interneuron Circuitry. Cell Rep..

[174] Bikoff J.B., Gabitto M.I., Rivard A.F., Drobac E., Machado T.A., Miri A., Brenner-Morton S., Famojure E., Diaz C., Alvarez F.J. (2016). Spinal Inhibitory Interneuron Diversity Delineates Variant Motor Microcircuits. Cell.

[175] Zholudeva L.V., Abraira V.E., Satkunendrarajah K., McDevitt T.C., Goulding M.D., Magnuson D.S.K., Lane M.A. (2021). Spinal Interneurons as Gatekeepers to Neuroplasticity after Injury or Disease. J. Neurosci..

[176] Cregg J.M., Leiras R., Montalant A., Wanken P., Wickersham I.R., Kiehn O. (2020). Brainstem neurons that command mammalian locomotor asymmetries. Nat. Neurosci..

[177] Ruder L., Schina R., Kanodia H., Valencia-Garcia S., Pivetta C., Arber S. (2021). A functional map for diverse forelimb actions within brainstem circuitry. Nature.

[178] Usseglio G., Gatier E., Heuze A., Herent C., Bouvier J. (2020). Control of Orienting Movements and Locomotion by Projection-Defined Subsets of Brainstem V2a Neurons. Curr. Biol..

[179] Mathis A., Mamidanna P., Cury K.M., Abe T., Murthy V.N., Mathis M.W., Bethge M. (2018). DeepLabCut: Markerless pose estimation of user-defined body parts with deep learning. Nat. Neurosci..

